# High interspecific competitiveness of the invasive plant *Xanthium italicum* Moretti severely reduces the yield and quality of *Carthamus tinctorius* L.

**DOI:** 10.1038/s41598-023-31101-0

**Published:** 2023-03-15

**Authors:** Xia Ma, Mingcai Hou, Miao Ma

**Affiliations:** 1 Key Laboratory of Xinjiang Phytomedicine Resource Utilization, Shihezi University, Shihezi, 832003 China; 2 Changji Environment and Ecology Bureau, Hutubi Branch, Hutubi, 831200 China

**Keywords:** Agroecology, Invasive species, Plant symbiosis

## Abstract

Safflower is an annual herb of Compositae, which has great economic value. To explore the impact of invasive weed *Xanthium italicum* Moretti on the economic crop safflower, field experiments were conducted, the growth-related characters and the relative intensity of competition between the two species was explored. The results showed that under monoculture conditions, the stem height, crown width, stem diameter and the biomass of *X. italicum* root, stem and leaves were 1.14, 1.96, 1.82, 4.42, 4.21 and 3.99 times as high as those of safflower, respectively. When the two species coexisted, the growth related characters of *X. italicum* were further significantly improved, while the growth related characters of safflower were significantly decreased. When coexisted with *X. italicum*, the corolla biomass, hydroxysafflor yellow A content of corolla, seed yields, 100-seed weight, and seed oil content of safflower in the interplanted treatment 90.04%, 33.11%, 63.89%, 40.58%, and 25.61% lower than those in the monocultured treatment, respectively. Relative yield (RY) and Competitive balance index (CB) of *X. italicum* and safflower showed that the interspecific competitiveness of *X. italicum* was significantly higher than that of safflower. Under the competitive inhibition of *X. italicum,* not only the vegetative growth, but also the reproductive growth, yield, and quality of the economic organs of safflower were significantly negatively impacted. Together, our findings provide important scientific basis for evaluating the invasion risks and consequences of safflower’s cropland ecosystem by *X. italicum*.

## Introduction

Biological invasion refers to a kind of species with a certain distribution and abundance in countries or regions outside its origin, capable of breeding offspring, and causing damage to natural communities and ecosystems in the invaded areas^1^. It has become one of the five major global environmental changes in the twenty-first century. Biological invasion not only destroys the original ecosystem of the invaded area, but also threatens local economic development^2–4^. Invasive plants, as an important group of invasive organisms^5^, assimilate the resources needed by local plants through complex competitions, which inhibits the survival and reproduction of local plants and poses a serious threat to local biodiversity ^6^.

*Xanthium italicum* was first discovered in China in 1991^7^, an annual invasive weed of Compositae native to North America, is widely distributed in America, Europe, Asia, and Oceania^8^. Due to its strong adaptability, high seed production, fast growth, and high dispersal ability, it is easy to form dominant species in the invaded community, resulting in the decline or even extinction of the native plants^9,10^. *X. italicum* has spread throughout many Chinese provinces such as Liaoning, Shandong, Hebei, Shaanxi, and Xinjiang presently^11,12^. It has brought serious harms to the local agricultural production, animal husbandry, and biodiversity, especially in Xinjiang.

Safflower (*Carthamus tinctorius* L.), a very important economic plant that could be used in food and manufacture dyes, is widely cultivated in Xinjiang^13,14^. The yields of dried safflower flowers and grains in Xinjiang account for more than 80% of their total outputs in China. Its dry corolla, rich in Hydroxysafflor yellow A, is a traditional Chinese medicinal material. It has the efficacy of treating dysmenorrhea, whooping cough, vitiligo, and psoriasis^15^. Besides, the linoleic and oleic acid is rich in its seeds, safflower seed oil is a kind of high-quality edible oil with many pharmacological activities such as anti-fibrosis, anti-diabetic, antitumor, anti-inflammatory, liver protection, anti-lipid, anti-coagulation, and anti-oxidation^16–20^.

After a plant invades a new habitat, they will gain advantages with native plants by occupying vacant ecological niches or through interspecific competition^21^. The invasive weed *X. italicum* has a high growth rate and is easy to form a dominant population in communities, while the farmland habitats usually have better water and nutrient resources^22^, so that *X. italicum* has a serious cover over the field crops, resulting in a serious crop yield reduction. However, *X. italicum* has spreaded into safflower fields and inhibited the growth of the crop making it a dwarf. There is no study on whether this phenomenon is caused by the interspecific competition of *X. italicum* at present. Therefore, a field experiment was designed in this study to investigate the interspecific competition between *X. italicum* and safflower^23^, aiming to explore whether *X. italicum* could inhibit the growth of safflower and decrease the yields and quality of its economic organs. We hypothesized that: (1) when coexisted, the interspecific competition of *X. italicum* might hinder the growth and reproduction of safflower; and (2) the interspecific competition of *X. italicum* might affect the yields and quality of the safflower’s economic organs (corollas and seeds). This study could provide experimental evidence for evaluating the impacts of *X. italicum* on the safflower.

## Result

### Comparison of morphological indicators between* X. italicum* and safflower

**Figure 1:**
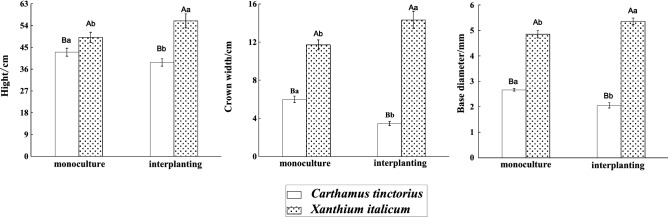
Morphological indicators of *X. italicum* and *Carthamus tinctorius* (mean ± SE). Different capital letters in the figure indicate significant differences between monoculture and interplanting treatments; different lowercase letters indicate significant differences between the two plants under the same planting pattern (*P* < 0.05).

Plants with similar ecological niches coexist, they often compete for limited environmental resources such as soil moisture, mineral nutrition and light in the environment. Plant size (height, crown width and base diameter) plays an important role in resource competition and is an important factor affecting growth rate ^24^. Morphological indicators is the most important index to reflect the good and bad of plant growth. The cultivating patterns had a great effect on the growth of *X. italicum* and safflower (*P* < *0.05*). Whether Under the interplanted condition or under the monoculture condition. *X. italicum* grows better than safflower (*P* < *0.05*) (Fig. 1). Under the monoculture condition, the height, crown width, and base diameter of *X. italicum* were 1.14, 1.96, and 1.82 times of safflower, respectively. Under the interplanted condition, the plant height, crown width, and base diameter of *X. italicum* were 1.44, 4.13 and 2.18 times as high as those of safflower (*P* < *0.05*). The plant height, crown width, and base diameter of *X. italicum* under the interplanted condition increased by 13.95%, 22.37%, and 10.49%, respectively compared with those under the monoculture treatment (*P* < *0.05*). However, the plant height, crown width, and base diameter of safflower under the interplanted condition decreased by 9.84%, 42.1% and 7.88%, respectively compared with those under the monoculture condition (*P* < *0.05*).

### Comparison of biomass between* X. italicum* and safflower

**Figure 2:**
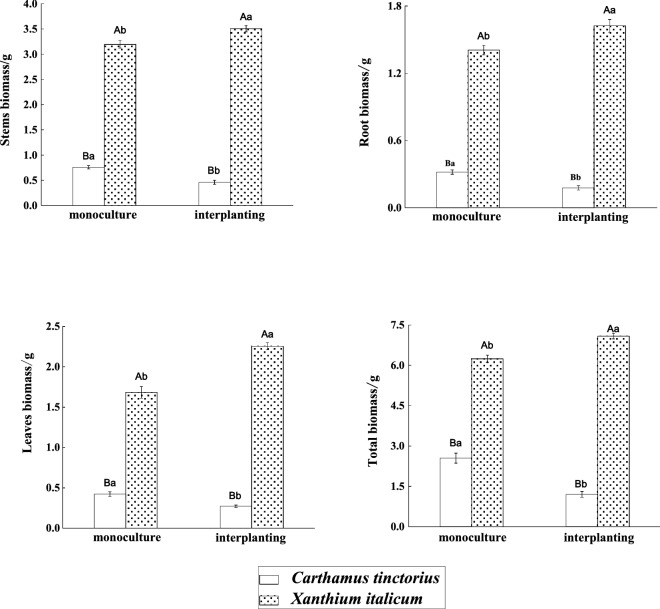
The biomass of *X. italicum* and *Carthamus tinctorius.*

Exotic plants tend to invade more successfully when there is lack of strong native resource competitors or when the community has sufficient resources available ^25^. Biomass is the most intuitive representation of good and bad plant growth. The cultivating patterns had a great effect on the growth of *X. italicum* and safflower (*P* < *0.05*) (Fig. 2). Whether Under the interplanted condition or under the monoculture condition. The total biomass of *X. italicum* was also higher than that of safflower (*P* < *0.05*). Under the monoculture condition, the biomass of roots, stems, leaves and total biomass of *X. italicum* were 4.42, 4.21, 3.99 and 2.45 times as high as those of the safflower, respectively *(P* < *0.05*). Under the interplanted condition, the biomass of roots, stems, leaves and total biomass were 9.17, 7.64, 8.29 and 5.89 times as high as those of safflower, respectively (*P* < *0.05*). And the biomass of roots, stems, leaves and total biomass of *X. italicum* under the interplanted condition increased by 15.35%, 9.7%, 33.99%, and 13.53%, respectively (*P* < *0.05*); while those of the safflower decreased by 44.34%, 39.53%, 35.55%, and 52.80%, respectively compared with those under the monoculture condition (*P* < *0.05*).

### Yields and quality of safflower economic organs

**Figure 3:**
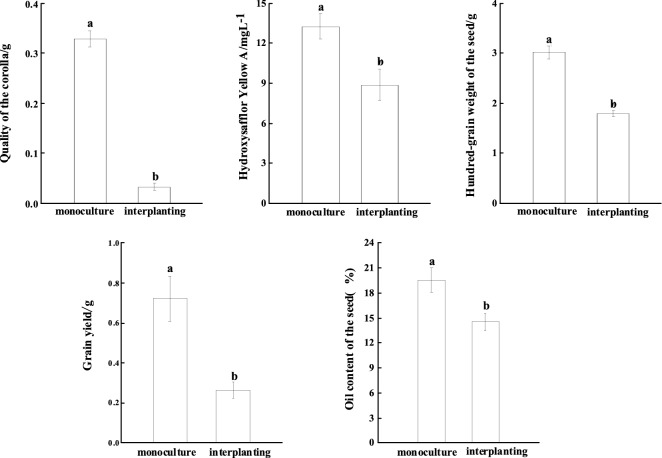
Yield and quality of the economic organs of *Carthamus tinctorius.*

Invasive weeds usually lead to a sharp decline in yield and quality of a crop for the competition of environmental resources ^26^. The corolla and grains are the most important economic organs of safflower. The corolla quality, grain yield, 100-seed weight, hydroxysafflor yellow A content, and seed oil content of safflower under the interplanted condition were decreased by 90.04%, 63.89%, 40.58%, 33.11%, and 25.61%, respectively (*P* < *0.05*) compared with those of safflower under the monoculture treatment (Fig. 3) planting pattern(*P* < *0.05*).

### Comparison of competitive ability between* X. italicum* and safflower

*RY* and *CB* were often used to characterize the niche similarity of two coexisting species and the relative intensity of their interspecific competitiveness ^27^. *RY* and *CB* were often used to characterize the niche similarity of two coexisting species and the relative intensity of their interspecific competitiveness. The *RY*_*X*_ were greater than 1.0, while *RY*_*C*_ was lower than 1.0 (*P* < 0.05) (Table 1), indicating that the intraspecific competition of *X. italicum* is significantly greater than the competition from the safflower, while the intraspecific competition intensity of safflower is significantly smaller than the competition from *X. italicum*. The *CBx* was greater than 0 and the *CBc* was lower than 0, indicating that the interspecific competitiveness of *X. italicum* is greater than that of safflower (*P* < 0.05) (Table 1).

**Table 1:** Competition index of *X. italicum and Carthamus tinctorius* under different treatments.

Relative yield of *Xanthium italicum*	Relative yield of *Carthamus tinctorius* L	Competitive balance index of *Xanthium italicum*	Competitive balance index of *Carthamus tinctorius L*
1.135 ± 0.037*	0.606 ± 0.018*	0.627 ± 0.056**	− 0.627 ± 0.056**

The values in the table are average values ± standard errors.

*RY_*X*_ and *RY*_*C*_ are compared with 1.

**CB_*X*_ is ompared with 0 (T-test:* P* < 0.05).

## Discussion

Competition commonly exists in plant communities. When plants with similar niches coexist, they often compete for limited environment resources such as soil moisture, mineral nutrition, and light^28^. Both *X. italicum* and safflower are shallow-rooted and sunny annual herbs of the Compositae family, with similar niches, so there is a potential interspecific competition between them. The results of this study showed that there were significant differences between the two species in terms of vegetative growth-related parameters: under monoculture condition, the plant height, crown width, base diameter, biomass of root, stem, and leaf of *X. italicum* were 1.14, 1.96, 1.82, 4.42, 4.21, 3.99 and 2.45 times as high as those of safflower, respectively. When the two species were interplanted, the difference in the vegetative growth-related parameters was more obvious. The plant height, crown width, base diameter, biomass of root, stem, leaf, and total biomass of *X. italicum* were 1.44, 4.13, 2.18, 9.17, 7.64, 8.29 and 5.89 times as high as those of the safflower, respectively. This may be due to the wider ecological range^10^, higher phenotypic plasticity, and rapider growth of *X. italicum* than those of safflower, which enable the alien plant *X. italicum* to develop higher aboveground organs, larger crown, more leaves, and larger leaf area than safflower in a short period of time. On the other hand, the intraspecific competition of *X. italicum* was significantly higher than the interspecific competition of safflower. Therefore, under interplanted condition, the difference in biomass between *X. italicum* and safflower was more significant, which makes *X. italicum* intercept more light from the canopy and reducing the use of light of the lower layer of safflower. It inevitably reduced the net photosynthetic rate and further biomass accumulation of safflower. In addition, a more developed root system means that the absorption capacity of *X. italicum* was higher than that of safflower, which enables *X. italicum* to obtain more water and mineral nutrition from soil when competing with safflower.

The plant height, crown width, base diameter, and biomass of *X. italicum* under interplanted condition were higher than that under the monoculture condition (*P* < 0.05), which means that the intraspecific competition intensity of the alien plant is significantly higher than the competition suppress from safflower. On the contrary, the plant height, crown width, base diameter, and biomass of safflower under the interplanted condition were lower than that under the monoculture condition (*P* < 0.05), indicating that the intensity of intraspecific competition of the safflower is lower than that of the alien species *X. italicum* (*P* < 0.05). *RY* and *CB* were often used to characterize the niche similarity of two coexisting species and the relative intensity of their interspecific competitiveness. This study showed that the relative yield of *X. italicum* was greater than 1.0, while that of safflower was lower than 1.0, indicating that the two species have similar niches (*P* < 0.05), and *X. italicum* has higher interspecific competitiveness than safflower (*CB*_*X*_ > 0). Therefore, *X. italicum* has a significant inhibition effect on the growth of safflower^29^. This may be the reason why the growth of safflower was suppressed after *X. italicum* was introduced into its habitates.

Invasive weeds usually lead to a sharp decline in yield and quality of a crop for the competition of environmental resources^30,31^. *X. italicum* is a highly competitive weed in farmland, and 8% of farmland coverage of *X. italicum* could cause crop yield loss of 60%. Our study showed that the corolla quality, grain yield, 100-seed weight, Hydroxysafflor yellow A concentration, and seed oil content of safflower were reduced by 90%, 64%, 41%, 33%, and 26% respectively as it coexisted with *X. italicum*. The dry corolla of safflower is a traditional Chinese medicinal material, and the Hydroxysafflor yellow A in the corolla is an active ingredient^32^. The sharp decline in the yield of corolla and the content of Hydroxysafflor yellow A means a sharp decline in the yield and quality of the medicinal material. Safflower seed is rich in oil. It is an important edible oil in the worldwide at present. Compared with interplanting, the hundred-grain weigh decreased by 48.48% of monoculture, which means that the seeds are significantly smaller and lighter. The sharp decline in seed yield and oil content means that its oil production decreased significantly, which will not only affect seed germination and seedling but also inevitably affect growth the economic benefits of farmers and oil extracting factories. The oil content of safflower seeds decreased by more than a quarter under interplanted condition with *X. italicum*, which directly affects the seed germination and the seedlings, survival, and has a potential negative impact on the yields of safflower in the next planting cycle.

## Conclusion

In summary, the ecological niches of *X. italicum* and safflower were similar. Therefore, there is always fierce interspecific competition for limited environmental resources between them. The interspecific competition of *X. italicum* is significantly higher than that of safflower, which not only significantly inhibits the individual vegetative growth of safflower and biomass accumulation, but also seriously inhibits its reproductive growth. When interplanted with *X. italicum*, the corolla yield, Hydroxysafflor yellow A concentration, seed production, and seed oil content of safflower decreased greatly. Therefore, the output and quality of safflower’s economic organs reduced severely.

## Materials and methods

### Plant material

One thousands of mature seeds of *X. italicum* with the same size were collected from the suburb of Shihezi, Xinjiang, China (44° 23′ N, 86° 00′ E) in October 2018, and one thousands of safflower (*Carthamus tinctorius* L.) (Yumin without spines) seeds with full grain and consistent size were collected from Yumin (45° 24′ E, 82° 12′ N) in Xinjiang, China in September 2018 (We obtained permission to collect seeds of *X. italicum* and *Carthamus tinctorius* L. from respective authority).

### Experimental design and method

A field experiment was conducted to explore the effect of interspecific competition of *X. italicum* and Safflower on the growth, yields, and quality of safflower’s economic organs. The trials were performed at the research farm of the Shihezi University (44° 19′ 0″ N, 86° 0′ 30″ E) from April 2019 to September 2019, with a temperate continental climate and annual precipitation ranging from 125.0 to 207.7 mm.

This study was designed as a randomized complete block design with three replications. Single sub-plot measured 6 m^2^ (2 m long × 3 m wide), on a sandy soil. The soil characteristics were: the total nitrogen content was 0.268 g/kg, total phosphorus content was 0.0855 g/kg, total potassium content was 5.72 g/kg, available nitrogen content was 43.59 mg/kg, available phosphorus content was 4.1 mg/kg, available potassium content was 119.09 mg/kg, and organic matter content was 5.81 g/kg.

Three treatments were involved in this experiment: monoculture of safflower, monoculture of *X. italicum*, and interplanting of the two (1:1). On April 6, 2019, *X. italicum* and safflower seeds were sowed soaked in plant spacing of approx 8 cm within row, and 25 cm between rows, in 50 plants/m^2^ density. Approximately a month after sowing, plants were thinned to achieve the targeted plant population. After emergence of four true leaves, fertilizers (CO(NH_2_)_2_: 35.312 g/m^2^, Ca(H_2_PO_4_)_2_·CaSO_4_·2H_2_O: 14.72 g/m^2^, and K_2_SO_4_: 11.776 g/m^2^) was applied according to the local practice^33^. The whole plants were harvested after the seeds matured.

### Data acquisition

#### Determination of morphological characters

Vertical height (cm) from the base to the top of plants were determined with a meter.

Crown width (cm) was determined with a meter through determining the maximum width and horizontal width of plants.

Crown width/cm refers to the mean of the maximum width of the plant and its horizontal width.

Base diameter (the diameter of stem 1 cm above the ground (mm) was determined with a meter.

### Determination of biomass

The root, stem, and leaf of *X. italicum* and safflower were separated and dried to constant weight in an oven at 70 ℃. The biomass of each part was obtained using an electronic balance (BS423S). The total biomass and root/shoot ratio were calculated with the following formulas.

$$\begin{aligned} & {\text{Total}}\;{\text{biomass}} = {\text{root}}\;{\text{biomass}} + {\text{stem}}\;{\text{biomass}} + {\text{leaf}}\;{\text{biomass}} + {\text{fruit}}\;{\text{biomass}} + {\text{corolla}}\;{\text{biomass}}. \\ & {\text{Root/shoot}}\;{\text{ratio}} = ({\text{root}}\;{\text{biomass/stem}}\;{\text{biomass}} + {\text{leaf}}\;{\text{biomass}}). \\ \end{aligned}$$

### Determination of yield and quality of safflower economic organs

The corollas of safflower were harvested after turning to red, and its biomass was weighed using an electronic balance (BS423S) after dried naturally in shade.

The dried safflower corollas were ground into a fine powder with a mortar and passed through an 80-mesh sieve. The powder of 0.040 g was weighed, transferred in a 10 mL centrifuge tube with 8 mL 25% methanol and extracted with an ultrasonic extractor (L6-180 LNB Instrument Co., Shanghai, China) for 30 min. The extract was centrifuged at 12,000 r/min for 10 min, and the supernatant was filtered with a 0.22 μm microporous filter. The filtrate was used for determining the concentration of Hydroxysafflor yellow A with Agilent High Performance Liquid Chromatography (HPLC) (Agilent 1200, Agilent Technologies, CA, USA).

The seeds of safflower were collected and dried naturally. After that, 100-seed weight and seed biomass were determined with an electronic balance (BS423S Sartorius, Beijing, China) and the average values were calculated. The oil content of the seeds was determined with a nuclear magnetic resonance spectrometer (CNMR-1000, Chenmu, Wuhan, China), and the average value was calculated based on 3 replicates.

#### Determination of interspecific competitiveness

To compare the interspecific competitiveness of the two species, relative yield (*RY*)^34^ and competitive balance index (*CB*)^35^ were calculated with the following formulas:

$${\text{R}}Y_{X} = Y_{{XC}} /Y_{X} ,RY_{C} = Y_{{CX}} /Y_{X}$$

$$CB_{X} = \ln ({\text{R}}Y_{X} /{\text{R}}Y_{C} ),CB_{C} = \ln ({\text{R}}Y_{C} /{\text{R}}Y_{X} )$$

where *X* is *X. italicum* and *C* is Safflower. *RY*_*X*_ and *RY*_*C*_ are the relative yield of *X. italicum* and safflower in the interplanted treatment, respectively. *Y*_*X*_ and *Y*_*C*_ are the average individual yield of *X. italicum* and safflower in the monocultured treatments, respectively. *Y*_*XC*_ and *Y*_*CX*_ are the average individual yield of *X. italicum* and safflower in the interplanted treatment, respectively.

If *RY*_*X*_ is greater than 1.0, the intraspecific competition of *X. italicum* is significantly greater than the interspecific competition between *X. italicum* and safflower. If *RY*_*X*_ is equal to 1.0, the competitiveness of the two species is equal. If *RY*_*X*_ is lower than 1.0, the intraspecific competition of *X. italicum* is significantly smaller than the interspecific competition between the two species.

If *CB*_*X*_ is greater than 0, the intraspecific competition of *X. italicum* is significantly greater than that the interspecific competition between *X. italicum* and safflower. The greater the *CB*_*X*_ value, the stronger the competitiveness of *X. italicum*. If *CB*_*X*_ is equal to 0, the competitiveness of *X. italicum* and safflower are equal but if *CB*_*X*_ is lower than 0, the competitiveness of *X. italicum* is significantly lower than that of the safflower. The smaller the *CB*_*X*_ value, the lower the competitiveness of *X. italicum*.

### Statistical analysis

SPSS software (version 20.0, USA) was adopted for statistical analyses. One-way ANOVA and nonparametric tests were used to compare the significance of the difference in growth indexes between *X. italicum* and safflower, and yield and quality of safflower economic organs between the monoculture and the interplanted treatments. The T-test was used to analyze the differences between the relative yield (*RY*) and 1, the competitive balance index (*CB*) and 0.

### Ethical statement

In our study, the studies of involving plants (*X. italicum* Moretti and *Carthamus tinctorius* L.) has been carried out in accordance with relevant institutional, national, and international guidelines and legislation.

## Data Availability

All the data presented in this manuscript. If Editorial Board Members and referees need data for the purposes of evaluating the manuscript, the original data can be provided by Xia Ma. Mingcai Hou and Xia Ma are responsible for the data.
